# Focus on the tumor microenvironment: A seedbed for neuroendocrine prostate *cancer*

**DOI:** 10.3389/fcell.2022.955669

**Published:** 2022-07-22

**Authors:** Hengfeng Zhou, Qiangrong He, Chao Li, Bassam Lutf Mohammed Alsharafi, Liang Deng, Zhi Long, Yu Gan

**Affiliations:** ^1^ Andrology Center, Department of Urology, the Third Xiangya Hospital, Central South University, Changsha, China; ^2^ Department of Urology, Xiangya Hospital, Central South University, Changsha, China

**Keywords:** neuroendocrine prostate cancer (NEPC), castration-resistant prostate cancer, tumor microenvironment, androgen deprivation therapy (ADT), lineage plasticity

## Abstract

The tumor microenvironment (TME) is a microecology consisting of tumor and mesenchymal cells and extracellular matrices. The TME plays important regulatory roles in tumor proliferation, invasion, metastasis, and differentiation. Neuroendocrine differentiation (NED) is a mechanism by which castration resistance develops in advanced prostate cancer (PCa). NED is induced after androgen deprivation therapy and neuroendocrine prostate cancer (NEPC) is established finally. NEPC has poor prognosis and short overall survival and is a major cause of death in patients with PCa. Both the cellular and non-cellular components of the TME regulate and induce NEPC formation through various pathways. Insights into the roles of the TME in NEPC evolution, growth, and progression have increased over the past few years. These novel insights will help refine the NEPC formation model and lay the foundation for the discovery of new NEPC therapies targeting the TME.

## Introduction

Prostate cancer (PCa) is the most common malignancy of the genitourinary system and in men worldwide. According to the US Center for Health Statistics, PCa ranks first and second in cancer incidence and mortality among males, respectively. PCa accounted for 27 and 11% of all new cancer cases and deaths, respectively, in the United States in 2022 (Siegel et al., 2022).

PCa is primarily a prostate adenocarcinoma (AD PCa). Therefore, androgen deprivation therapy (ADT) is the first-line treatment for it (Huggins and Hodges, 1972; Koutsilieris et al., 2006; Davies et al., 2018; Yu et al., 2018). Clinical studies have shown that ADT has therapeutic efficacy for most patients in the early stages of PCa. Nevertheless, PCa gradually adapts to low testosterone levels and eventually progresses to castration-resistant prostate cancer (CRPC) (Mizokami and Namiki, 2015; Wade and Kyprianou, 2018; Wu et al., 2019). The median survival time for CRPC is only 15–30 months and the disease responds poorly to traditional anti-androgen receptor (AR) drugs such as bicalutamide (Tran et al., 2009; Conteduca et al., 2019). Thus, next-generation AR pathway inhibitors (ARPIs) such as enzalutamide and abiraterone have been developed. Patients with PCa may nonetheless acquire resistance to ARPIs. Neuroendocrine differentiation (NED) occurrence and neuroendocrine prostate cancer (NEPC) formation are vital mechanisms of resistance to ARPIs(Long et al., 2021). Essentially, NEPC is a representative type of recurrent tumor with lineage change developing under the survival pressure from targeted therapies. The expression of the AR receptor through which the PCa tumor is treated is repressed in this setting. Consequently, ARPIs cannot inhibit tumor growth because tumors lose their dependence on AR signaling (Nouri et al., 2014; Vlachostergios et al., 2017; Beltran et al., 2020; Bhagirath et al., 2020). At the clinical level, tumor cells proliferate and grow independently of the AR signaling pathway. NEPC may undergo visceral and osteolytic metastasis. NEPC tumors express neuroendocrine (NE)-related markers such as chromogranin A (CHGA), neuron-specific enolase (NSE), synaptophysin (SYP), and CD56 (Dardenne et al., 2016; Pifano et al., 2017; Conteduca et al., 2019). NEPC prognosis is poor and its OS is <1 year. NEPC therapy remains a challenge as the mechanism of its formation is unclear.

### Current research suggests the following putative neuroendocrine prostate cancer formation mechanisms

1) Cancer-like stem cell model

Lineage plasticity is the ability of cells to transition from one committed developmental pathway to another (Lee et al., 2018; Gan et al., 2018; Lovnicki et al., 2020; Quintanal-Villalonga et al., 2020; Yang et al., 2022). Cells can be reprogrammed and pushed back to a state like that of stem cells and then differentiated. Epithelial tumor cells undergo epithelial-mesenchymal transition (EMT). The former acquire stem cell properties and dedifferentiate into tumor-like stem cells (CSCs). Under androgen deprivation, CSCs differentiate into neuroendocrine cells (NECs), which, in turn, develop into NEPC. The tumor cell dedifferentiation/redifferentiation process is known as NED (Meacham and Morrison, 2013; Rich, 2016; Davies et al., 2018; Beltran et al., 2019). Lineage plasticity plays an important role in the CSC model.

2) Hierarchy differentiation model

A few neuroendocrine tumor cells occur in AD PCa and originate from two sources. Normal stem cells in the prostate undergo cancer-derived mutations and are transformed into CSCs, which, in turn, differentiate into epithelial or neuroendocrine tumor cells. Normal NECs undergo cancerous mutations and are transformed into neuroendocrine tumor cells. The number of epithelial tumor cells decrease under androgen deprivation. Neuroendocrine cells rapidly proliferate, predominate, and complete the process of AD PCa transformation to NEPC (Davies et al., 2018). However, the foregoing models do not fully account for NEPC formation. The mainstream view is that both mechanisms complement each other and collaborate to promote NEPC formation. Recent studies have indicated that the tumor microenvironment (TME) plays critical roles in both models of NEPC formation (Kim and Zhang., 2016; Long et al., 2020; Mehraj et al., 2021).

The TME is the internal and external tumor cell environment and includes the structure, function, and metabolism of the tissue wherein the tumor is located. It is closely related to tumor occurrence, growth, metastasis, and cell differentiation and has both cellular and non-cellular components. The cellular components include the tumor cells, immune cells, cancer-associated fibroblasts (CAFs), and vascular endothelial cells (VECs). Through various pathways, they form a complex intercellular signaling network, shape the extracellular matrix (ECM), and control angiogenesis. The non-cellular components include the ECM and inflammatory factors, chemokines, and matrix enzymes. They promote tumor evolution, growth, and progression by mediating intercellular signaling. Tumor growth and infiltration require interactions between tumor cells and the surrounding microenvironment. Tumor cells induce TME remodeling to protect themselves from apoptosis and stimulate angiogenesis (Wade and Kyprianou, 2018; Wu et al., 2018; Bonollo et al., 2020).

The TME of NEPC is highly heterogeneous and characterized by abnormal tumor metabolism, hypoxia, necrosis, and massive mitosis (Ippolito et al., 2016; Cimadamore et al., 2020). Both the cellular and non-cellular components of the TME play crucial roles in NEPC formation and maintenance (Dicken et al., 2019). *De novo* NEPC occurs in <1% of all patients with PCa. After ADT, however, ∼10–15% of all patients with PCa develop tumors with NE features. Therefore, the effects of androgen deprivation on the TME may induce NEPC(Nouri et al., 2014; Aggarwal et al., 2017; Davies et al., 2018; Gan et al., 2018; Beltran et al., 2019; Kato et al., 2019).

There are no specific therapeutic options for NEPC. Hence, suppressing EMT and NED in PCa has become a research hotspot. The TME promotes tumor cell differentiation and induces EMT and NED in response to androgen deprivation (Rich, 2016; Tu et al., 2021; Zheng et al., 2021). In recent years, recognizing and regulating the interactions between the TME and NEPC have become focal points in prostate cancer research. In the present review, we discuss the characteristics of the NEPC-related TME.

### Cellular components in the neuroendocrine prostate cancer microenvironment

#### Prostatic epithelial cells

In AD PCa, prostatic epithelial cells are the most dominant cell subset in the TME, thus playing an important role in the development of NEPC.

On the one hand, NEPC is essentially developed through the transformation of prostatic epithelial cell lineage into NECs. This process is regulated by many genes, such as Forkhead Box Protein C2 (FOXC2). FOXC2 is a transcription factor (TF) known to promote cancer stemness and metastasis. Moreover, it can induce NEPC development (Paranjape et al., 2016). Other genes, such as MYCN and BRN2, also play an important role in the process of prostatic epithelial cell lineage transition (Chen et al., 2018; Davies et al., 2018).

On the other hand, prostatic epithelial cells can regulate other components of the TME, indirectly promoting NEPC development. For example, prostatic epithelial cells secrete bone morphogenetic protein-6 (BMP-6) and cyclooxygenase 2 (COX2), which promotes the transformation of TAMs from the M1 to the M2 subtype (Heusinkveld et al., 2011; Lee et al., 2011).

### Immune cells

Immune cells are essential components of the human defense system. They perform immune surveillance and detect and remove tumor cells in the early stages of tumor formation. As cancer progresses, however, tumor cells may avoid the lethal effects of immune cells via immune evasion, immune tolerance, and other mechanisms. Tumor cells can even induce immune cell infiltration. Long-term immune cell infiltration promotes tumor growth and development. Certain tumor-infiltrating immune cells such as TAMs and myeloid-derived suppressor cells (MDSCs) participate in NEPC formation (Leone and Powell, 2020; Zhao et al., 2021). The summary of the roles of cellular components can be seen in Table 1.

**TABLE 1:** Role of cellular components in the NEPC microenvironment.

Cellular components	Cytokine	Recruitment factor	References
M2-TAMs	IL-6	HMGB-1, BMP-6, PGE2, CSF-1, CCL2	Roca et al. (2009); Lee et al. (2011); Lin et al. (2013); Jia et al., (2014); Leone and Powell. (2020); Zhong et al. (2021)
MDSCs	IL-6	IL-6-STAT3	Lopez-Bujanda and Drake, (2017); Moreira et al. (2018); Hellsten et al. (2019); Koinis et al. (2021); Mukherjee et al. (2021)
CAFs	SFRP-1-MYCN/AURKA, RASAL3		Kato et al. (2019); Nguyen et al. (2019); Bonollo et al. (2020)
MSCs	TGF-β1, CCL5		Ye et al. (2012); Ridge et al. (2017); Yu et al., (2018)
VECs		FAP, MMP	Chien et al. (2001); Hulsurkar et al. (2017); Wang et al. (2019)

### TAMs

TAMs are infiltrating macrophages in tumor tissue and differentiate mainly from monocytes. They are recruited by chemotactic signals such as colony-stimulating factor-1 (CSF1) and chemokine (C-C motif) ligand-2 (CCL2) released by the tumor and/or non-tumor cells in the TME (Roca et al., 2009; Lin et al., 2013; Leone and Powell, 2020). In the early stages of tumor formation, the M1 subtype predominates among TAMs, killing tumor cells through nitric oxide (NO). As tumors progress, however, the M1 TAMs transform into the M2 subtype, promoting tumor development and inducing NED in various ways (Chen et al., 2019; Leone and Powell, 2020).

Chao Wang et al. found that M2 TAMs play essential roles in enzalutamide-induced NED (Wang et al., 2018). Enzalutamide upregulates high mobility group protein B1 (HMGB1) in the tumor cell cytoplasm. HMGB1 is an essential late-stage inflammatory factor that recruits macrophage infiltration and activates NED induction by macrophages (Ellerman et al., 2007; Jia et al., 2014). Chao Wang et al. co-cultured monocytes with androgen-independent enzalutamide-treated C4-2 cells derived from the LNCaP sub-line and found that interleukin-6 (IL-6) secretion by monocytes and NSE and CHGA expression in progeny cells were increased. Anti-HMGB1 monoclonal antibodies significantly reduced IL-6 secretion during co-culture. Hence, IL-6 is secreted by M2-TAMs recruited by HMGB1 (Wang et al., 2018). The addition of IL-6 receptor inhibitors during co-culture prevented NE marker upregulation in progeny cells. Thus, tumor-infiltrating M2-TAMs promote the NED process by activating downstream IL-6 signaling (Wang et al., 2018; Zhong et al., 2021).

Lee and Geun Taek reported that BMP-6 secreted by PCa cells induces M2-TAMs to secrete IL-6. This finding confirms the cellular network between PCa cells and TAMs (Lee et al., 2011).

### Myeloid-derived suppressor cells

Myeloid-derived suppressor cells (MDSCs) constitute a highly heterogeneous cell population derived from bone marrow. They consist mainly of immature macrophages, dendritic cells, and granulocytes and have immunosuppressive activity (Kohada et al., 2021). MDSCs inhibit T cell activation, prevent dendritic cell maturation, and induce natural killer (NK) cell anergy (Kohada et al., 2021; Koinis et al., 2021).

Current research on MDSCs in PCa has focused primarily on immune evasion and the promotion of CRPC formation. However, certain molecular signaling pathways in MDSCs are implicated in NED occurrence. Rebecka Hellsten et al. stated that the signal transducer and activator of transcription (STAT3) inhibitor galiellalactone prevents MDSC aggregation. Thus, activation of the STAT3 signaling pathway may be critical to MDSC recruitment. The recruited MDSCs induce NED by secreting IL-6 and activating downstream STAT3 signaling. Therefore, STAT3 plays essential roles in both MDSC recruitment and NED induction by MDSCs(Moreira et al., 2018; Hellsten et al., 2019; Koinis et al., 2021; Mukherjee et al., 2021). Toll-like receptor 9 (TLR9) signaling in PCa cells stimulates STAT3 signaling downstream of MDSCs and causes the latter to induce NED (Hossain et al., 2015; Moreira et al., 2018; Koinis et al., 2021).

MDSCs also induce monocytes to differentiate into M2-TAMs via paracrine IL-6 and promote naive T cell differentiation into T cell subtypes that secrete abundant IL-17 (Roca et al., 2009; Heusinkveld et al., 2011; Lopez-Bujanda and Drake, 2017). IL-17 causes PCa cells to secrete COX-2, promoting the conversion of arachidonic acid into prostaglandin E2 (PGE2) and inducing monocyte differentiation into M2-TAMs, which activate downstream pathways and induce NED (Heusinkveld et al., 2011; Lopez-Bujanda and Drake, 2017).

### Cancer-associated fibroblasts

Cancer-associated fibroblasts (CAFs) are various tumor-related matrix cells. They are the most abundant cells in the TME and significantly contribute to tumor migration, invasion, immune escape, and ECM remodeling (Bonollo et al., 2020). CAFs may promote NEPC formation by a direct pathway or via the Ras protein activator-like 3 (RASAL3) epigenetic silencing pathway (Nguyen et al., 2019; Bonollo et al., 2020).

Kato et al. mentioned that CD105^+^ CAFs induce NED by initiating the paracrine SFRP1 signaling axis (Kato et al., 2019). CD105 (endoglin) is a transforming growth factor beta (TGF-β)-type III receptor that promotes BMP signaling and inhibits TGF-β signaling (Fonsatti et al., 2003). CD105^+^ CAFs initiate the paracrine SFRP1 signaling axis and upregulate *MYCN* and *AURKA* expression, which promotes NEPC formation. TRC105 (humanized CD105 neutralizing antibody) downregulates SFRP1 in fibroblasts and inhibits NED induction (Kato et al., 2019).

Rajeev Mishra et al. showed that the proto-oncogene Ras suppressor RASAL3 is epigenetically silenced through the methylation of the CpG island in *RASAL3*. Stable AR signaling prevents methylation-induced epigenetic *RASAL3* silencing. Thus, ADT promotes epigenetic *RASAL3* silencing and enhances Ras activity (Mishra et al., 2018; Li et al., 2021). Ras protein induces phagocytosis in CAFs and hydrolyzes albumin to glutamine, which is released into the TME and transformed into PCa epithelial cells. Glutamine upregulates *FOXA2, AURKA*, and other genes, and induces NED by activating the mammalian target of rapamycin (mTOR) (Kanayama et al., 2017; Mishra et al., 2018).

### Mesenchymal stem cells

Mesenchymal stem cells (MSCs) are pluripotent stem cells that differentiate into osteoblasts, chondrocytes, and adipocytes and are positive for the CD73, CD105, and CD90 surface markers (Dominici et al., 2006; Ridge et al., 2017). A few studies revealed that MSCs may increase cell stemness and contribute to tumor progression (Kansy et al., 2014; Ridge et al., 2017).

MSCs promote EMT in breast, gastric, and liver cancer cells. EMT transforms AD PCa into NEPC. Therefore, MSCs may be implicated in NEPC formation (Karnoub et al., 2007; Jing et al., 2012; Kansy et al., 2014; Xue et al., 2015). Under ADT, MSCs increase PCa cell stemness by promoting CCL5 secretion and inducing EMT (Yu et al., 2018). Yang Yu et al. co-cultured MSCs with PC3 cells (a human prostate cancer cell line) *in vitro* and discovered that TGF-β1 secretion increased in the culture matrix in a time-dependent manner. TGF-β1 activates downstream signaling pathways to induce NED. ThusMSCs also promote NEPC formation (Ye et al., 2012; Ridge et al., 2017; Yu et al., 2021).

### Vascular endothelial cells and angiogenesis

Vascular endothelial cell (VEC) proliferation and local angiogenesis in the tumor are reactively promoted. Tumor cells require abundant oxygen and metabolites for rapid proliferation (Deep and Panigrahi, 2015; Wang et al., 2021). Certain targets that promote angiogenesis also promote NED.

Yan Zhang et al. disclosed that PCa cells treated with enzalutamide activate enhanced zeste homolog 2 (EZH2) via the cAMP-response element binding protein (CREB) signaling pathway. Subsequently, EZH2 inactivates various genes by trimethylation at Lys 27 of histone H3 (H3K27me3) and promotes NED (Dardenne et al., 2016; Zhang et al., 2018). Thrombospondin-1 (TSP1) prevents vascular endothelial growth factor (VEGF) from combining with its receptor and inhibits angiogenesis. In PCa, TSP1 is inhibited as a downstream target of CREB/EZH2 (Hulsurkar et al., 2017; Wang et al., 2019). Therefore, the CREB/EZH2/TSP1 pathway may be a common target of angiogenesis and NED (Zhang et al., 2018; Wang et al., 2019).

Calcitonin is another target linking angiogenesis and NED. It regulates neuroendocrine tissue function and is overexpressed in NEPC(Deftos, 1998; Chien et al., 2001). Srinivasulu Chigurupati et al. reported that calcitonin promotes human microvessel endothelial cell (HMEC-1) proliferation and induces HMEC-1 to form a microvascular network (Chigurupati et al., 2005). However, it remains to be determined how calcitonin promotes NEPC formation.

### Non-cellular components in the neuroendocrine prostate cancer microenvironment

#### Inflammatory factors

**FIGURE 1:**
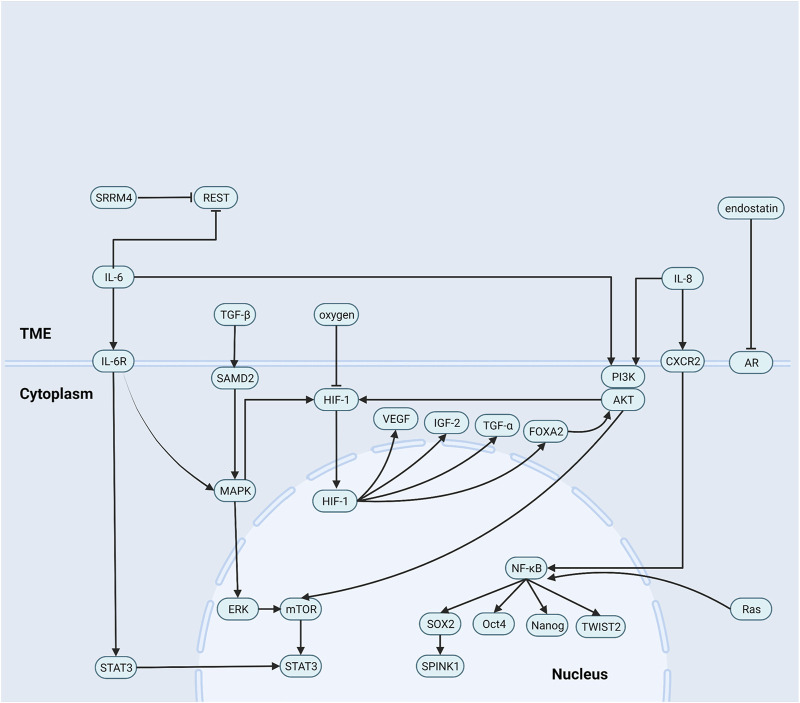
Role of non-cellular components in the NEPC microenvironment. (1) IL-8 activates NF-κB pathway signaling to increase levels of a series of stem cell transcription factors expression. (2) IL-6 induces neuroendocrine prostate cancer formation by activating STAT3 and MAPK/ERK signaling, and reducing the REST. Besides, IL-8 and IL-6 upregulate HIF-1 via PI3K/AKT/HIF-1α pathway and MAPK/HIF-1α pathway. (3) TGF-β/SMAD2 pathway signaling activates p38 MAPK and then induces neuroendocrine differentiation. (4) endostatin blocks androgen receptor signaling and then promotes the formation of epithelial tumor cells into androgen-independent cells.

Previous studies confirmed that tumors recruit and activate immune cells and that inflammatory factor concentrations significantly increase in the TME (Singh et al., 2019; Khandia and Munjal, 2020). Inflammatory factors transmit signals promoting androgen-independent (AI) cells self-renewal and proliferation. AI cells are important bridges in NEPC formation, autonomously secrete the proinflammatory factors IL-8, IL-6, and TGF-β, protect themselves against apoptosis, and induce NED (Archer et al., 2020; Kiely and Ambs, 2021; Zhong et al., 2021). The summary of the roles of non-cellular components can be seen in Figure 1.

### IL-8

IL-8 is a vital inflammatory response factor that regulates tumor behavior, reduces LNCaP cell (an AR-dependent prostate cancer cell line) sensitivity to ADT, downregulates prostate-specific antigen (PSA) and AR, and activates the nuclear factor kappa-light-chain-enhancer of activated B cells (NF-κB) signaling pathway.

NF-κB induces stemness and lineage transformation in pancreatic and skin cancers (Liu Z. et al., 2021). In PCa, NF-κB upregulates the stem cell TFs TWIST2, SOX2, Oct4, and Nanog, which control the numbers of cancer stem cells (CSCs) (Thomas-Jardin et al., 2020; Zhong et al., 2021). SOX2 causes prostate cancer cells to dedifferentiate into CSCs and activate the proto-oncogene SPINK1, which induces CSC differentiation into neuroendocrine tumor cells (Bhatia and Ateeq, 2019; Tiwari et al., 2020; Yamada and Beltran, 2021).

IL-8 stimulates CXC chemokine receptor 2 (CXCR2) via autocrine signaling to recruit immune cell infiltration and induce NED (Huang et al., 2005; Li et al., 2019). Kim et al. reported that blocking CXCR2 reduces the number of AI cells. The foregoing findings provide additional evidence that IL-8 promotes NED (Kim et al., 2017).

### IL-6

IL-6 induces NEPC formation by activating STAT3 and MAPK/ERK signaling and downregulating the RE-1 silencing transcription factor (REST). It promotes Tyr residue phosphorylation in the IL-6 receptor gp130. Phosphorylated gp130 then serves as a TF upregulating STAT3. IL-6/STAT3 pathway signaling upregulates VEGF and other cytokines and recruits various immune cells to infiltrate and control the numbers of CSCs(Drost and Agami, 2009; Iliopoulos et al., 2009; Iliopoulos et al., 2010; Iliopoulos et al., 2011; Kim et al., 2017). IL-6 activates MAPK/ERK signaling and upregulates MYCN and STAT3, which contribute to NEPC formation (Kim et al., 2017). Yezi Zhu et al. found that IL-6 decomposes REST by ubiquitination (Zhu et al., 2014). REST silencing is a marker of NEPC(Svensson et al., 2014; Beltran et al., 2019). Ruiqi Chen et al. demonstrated that REST degradation directly inhibits PI3K/AKT signaling and upregulates SYP in tumor cells (Schoenherr and Anderson, 1995; Zhang et al., 2015; Chen et al., 2017).

Both IL-6 and IL-8 upregulate hypoxia-inducible factor 1 (HIF-1α) via the PI3K/AKT/HIF-1α and MAPK/HIF-1α pathways to promote NEPC formation (Maxwell et al., 1999; Jaakkola et al., 2001).

### TGF-β

In the early stages of tumor development, TGF-β induces apoptosis and inhibits tumor cell proliferation. In the later stages of tumor development, it promotes tumors by regulating genomic instability and inducing EMT, angiogenesis, and immune evasion.

In breast and cutaneous squamous cell carcinomas, the TGF-β/SMAD pathway is associated with tumor formation and controls the numbers of CSCs(Massagué, 2012; Pang et al., 2018; Najafi et al., 2019). In hepatocellular carcinoma, TGF-β1 induces CD133 promoter demethylation by downregulating DNA methyltransferases 1 (DNMT1) and 3b (DNMT3b). The demethylated CD133 promoter is characteristic of a multi-lineage transformation ability (You et al., 2010). Therefore, TGF-β may also induce lineage transition and NED and promote NEPC formation (Dicken et al., 2019).

Haley Dicken et al. mentioned that the interaction between the TGF-β signaling network and the AR signaling axis induces EMT and enables epithelial-derived PCa tumor cells to acquire the NE phenotype (Dicken et al., 2019). Sirisha Natani et al. stated that IL-6 induces massive TGF-β secretion, while TGF-β/SMAD2 signaling activates p38 MAPK and induces NED (Natani et al., 2022). Thus, TGF-β receptor signaling-targeting therapy could inhibit both EMT and NED (Dicken et al., 2019).

### Exosomes

In PCa, CAFs secrete exosomes that transport paracrine signals such as micro RNAs (miRNAs) into the TME (Ishii et al., 2018). MiRNAs are small non-coding RNAs ∼20–23 nucleotides in length that downregulate specific tumor suppressors, proto-oncogenes, and other genes at both the transcriptional and translational levels. MiR-146a-5p inhibits epidermal growth factor receptor (EGFR) expression, angiogenesis, tumor proliferation, and migration (Ishii et al., 2018). Several miRNAs are implicated in NEPC formation. However, only a few of them have positive or negative regulatory functions. MiR-194 and miR-37 promote NEPC formation, while X chromosome miRNA clusters and miR-146a-5p inhibit it. Nevertheless, the mechanisms of other miRNAs remain to be elucidated. The summary of the roles of miRNAs can be seen in Table 2.

**TABLE 2:** Exosome-associated miRNAs and their functional relevance in NEPC.

miRNAs	Effects on NEPC	Related genes	References
miR-194	Promote	SOCS2, STAT3, ERK, FOXA1, Bmi-1	Huang et al. (2016); Das et al. (2017); Fernandes et al. (2021)
miR-375	Promote	PI3K/AKT, NCAM	Liu et al. (2013); Lin et al. (2013); Bhagirath et al. (2020)
chromosome 13 miRNAs cluster	Promote	SOX4, RB1, TP53, PTEN	Liang et al. (2011); Mogilyansky and Rigoutsos (2013); Wang et al. (2013); Fan et al. (2014); Tan et al. (2014); Hu et al. (2017); Liu et al. (2019)
miR-106b	Promote	RB1, TP53, PTEN, REST, EZH2	Cai et al., (2011); Liang et al., (2014)
miR-32, miR-221	Promote	RB1, PTEN	Mercatelli et al. (2008); Garofalo et al. (2009); Lupini et al. (2013)
X chromosome miRNAs cluster	Inhibit	AURKA, STAT3, MYCN, E2F1, PI3K/AKT, TP53, RB1, PTEN	Liang et al., (2011); Wang et al., (2013); Arabi et al., (2014); Fan et al. (2014)
miR-146a-5p	Inhibit	EGFR/ERK	Zhang et al. (2020); Zhao et al. (2020); Fan et al. (2014)

### MiRNAs promoting neuroendocrine prostate cancer formation

MiR-194 is negatively correlated with AR activity (Fernandes et al., 2021). MiR-194 downregulates the suppressor of cytokine signaling 2 (SOCS2) by ubiquitination and increases the concentrations of the key STAT3 and ERK pathway enzymes JAK2 and FLT3. In this manner, miR-194 induces EMT and promotes NEPC formation (Das et al., 2017). MiR-194 promotes NED by reducing FOXA1 secretion and upregulating Bmi-1 (Huang et al., 2016; Fernandes et al., 2021). FOXA1 induces tumor cell proliferation by activating the AR pathway but downregulates IL-8 by binding to its promoter and inhibiting EMT. Therefore, FOXA1 deletion is a critical NED marker (Kim et al., 2017). The proto-oncogene Bmi-1 causes CSCs to renew and control the numbers of CSCs(Huang et al., 2016).

MiR-375 plays roles in neuroendocrine tissues and induces tumor cells to express SYP, NSE, and CHGA(Bhagirath et al., 2020; Bhagirath et al., 2021). MiR-375 downregulates the tumor suppressor gene *TP53* in gastric neuroendocrine tumors. It also promotes neuronal cell growth and proliferation in neural tissue by upregulating neural cell adhesion molecule (NCAM) (Abdelmohsen et al., 2010; Liu et al., 2013). In PCa, the PI3K/AKT pathway induces NED, and miR-375 upregulates AKT (Bhagirath et al., 2020).

The oncogenic miRNA cluster on human chromosome 13 includes miR-17, miR-18a, miR-19a, miR-20a, miR-19b-1, and miR-92a-1 (Mogilyansky and Rigoutsos, 2013), downregulates the tumor suppressor genes *PTEN, TP53,* and *RB1*, and upregulates the TF SOX4. The latter induces the expression of pan-neural genes and regulates neuronal growth (Liang et al., 2011; Wang et al., 2013; Arabi et al., 2014; Fan et al., 2014; Tan et al., 2014; Hu et al., 2017; Liu et al., 2019; Liao et al., 2020).

MiR-106b, miR-32, and miR-221 downregulate *PTEN, TP53,* and *RB1* and increase the probability of tumor cell mutation (Mercatelli et al., 2008; Garofalo et al., 2009; Cai et al., 2011; Kumar et al., 2011; Lupini et al., 2013). MiR-106b downregulates REST and induces NED (Liang et al., 2014).

### MiRNAs inhibiting neuroendocrine prostate cancer formation

The miRNA cluster on the human X-chromosome includes miR-106a, miR-18b, miR-19b, miR-20b, miR-92a, and miR-363. Downregulation of the X-chromosome miRNA cluster upregulates *CHGA* and *SYP*(Bhagirath et al., 2020). The X-chromosome miRNA cluster activates the PI3K/AKT pathways and upregulates *PTEN, TP53,* and *RB1* (Fang et al., 2013; Arabi et al., 2014; Fan et al., 2014; Liao et al., 2020). Hence, it may prevent NEPC formation by inhibiting NED.

MiR-146a-5p inhibits NEPC formation by suppressing the expression of EGFR. EGFR signaling induces NEPC formation by promoting angiogenesis and gene mutation while downregulating *PTEN* (Zhang et al., 2020). ErbB2 is a member of the EGFR family that controls the numbers of CSCs, inhibits their apoptosis, and promotes NEPC formation (Standop et al., 2005). Stable AR binds androgen response element 2 (ARE2) and upregulates miR-146a-5p in hepatocellular carcinoma (Zhang et al., 2020; Zhao et al., 2020). Therefore, ADT may upregulate EGFR signaling and promote NEPC formation by downregulating miR-146a-5p.

### Hypoxia

Rapid tumor cell proliferation and a paucity of blood vessels may lead to localized hypoxia within the tumor. Hypoxia induces EMT in pancreatic, colon, and breast cancer. The relationship between hypoxia and EMT has been empirically demonstrated (Li et al., 2016).

HIF-1 is activated in the hypoxic microenvironment and controls the expression of genes associated with angiogenesis, EMT, stemness, and castration resistance (Skvortsov et al., 2018; Tao et al., 2021). HIF-1 comprises one α-subunit and one β-subunit. HIF-1α is inactivated by the oxygen-dependent ubiquitin-activating enzyme E3, and hypoxia inhibits its inactivation by preventing the ubiquitin-proteasome pathway. HIF-1 enters the nucleus, combines with hypoxia response element (HRE), and upregulates FOXA2. The latter upregulates *MYCN* and *AKT1* and promotes NEPC formation (Qi et al., 2010; Chen et al., 2018; Liu Q. et al., 2021; Berchuck et al., 2021).

HIF-1 also upregulates VEGF, insulin-like growth factor 2 (IGF-2), and TGF alpha (TGF-α), thereby indirectly promoting NEPC formation (Bresciani et al., 2019). All aforementioned growth factors promote cell proliferation. Each tumor cell division is associated with numerous errors in genes and protein synthesis that result in the loss of *P53, RB1*, and *PTEN,* which, in turn, increases the probability of NEPC formation (Zhao et al., 2018; Toth et al., 2019).

### Extracellular matrix

The ECM is a ubiquitous non-cellular component that provides mechanical support for cells and regulates cell proliferation, differentiation, migration, and lineage conversion (Egeblad and Werb, 2002; Cox, 2021).

The ECM is remodeled by fibroblast activation protein (FAP) and matrix metalloproteinases (MMPs) that are co-secreted by CAFs and tumor cells. FAP and MMPs are regulated by tissue inhibitors of metalloproteinases (TIMPs). The MMP/TIMP system is the most crucial enzyme system in ECM remodeling. In PCa, MMPs are generally upregulated, TIMPs are downregulated, and the ECM induces angiogenesis and EMT (Chantrain et al., 2004; Littlepage et al., 2010). Laurie E. Littlepage et al. discovered that MMP-2, MMP-7, and MMP-9 were significantly upregulated in the AR-negative, androgen-independent PCa CR2-TAg cell line expressing NE markers. Thus, the ECM may promote NEPC formation (Lee et al., 2015).

A portion of the ECM contains recessive domains that structurally resemble certain chemokines and cytokines. When the ECM is hydrolyzed, its recessive domains are activated and behave like chemokines and cytokines (Ricard-Blum and Vallet, 2016). Endostatin is an ECM component that downregulates VEGF and inhibits blood vessel formation. In PCa, endostatin blocks AR signaling and promotes the transformation of epithelial tumor cells into AI cells (Ricard-Blum and Vallet, 2016; Cox, 2021).

### Tumor cell density

Zuzana Pernicová et al. reported that when LNCaP and C4-2 cells are co-cultured at 20,000/cm^2^ in the presence of androgen, NED occurs in both modes. Therefore, the mechanism of cell density-induced NED may not be associated with the inhibition of AR activity (Pernicová et al., 2014). Zuzana Pernicová et al. also discovered that in both modes, G1 phase arrest occurred and was mediated through the inhibition of the cell cycle regulators CDK1 and CDK2 (Knudsen et al., 1998; Balk and Knudsen, 2008; Pernicová et al., 2014). Thus, CDK1 and CDK2 might promote cell density-controlled NEPC formation.

High cell densities activate cyclic adenosine 3′,5′-monophosphate (cAMP) signaling and upregulate cAMP-dependent protein kinase A regulatory subunit 2 (PKA RII). The cAMP signaling phosphorylates cAMP response element binding (CREB) protein and induces NED (Bang et al., 1994; Cox et al., 2000; Kregel et al., 2013). When cAMP signaling is inhibited with the specific adenylate cyclase inhibitor MDL-12330A, the percentage of cells that exert NED is significantly reduced. This phenomenon demonstrates that high cell density promotes NEPC formation via the cAMP pathway (Pernicová et al., 2014).

### Androgen deprivation therapy and the tumor microenvironment

NEPC formation is associated with castration resistance (Ferrari et al., 2017). The following subsection addresses the mechanisms by which ADT affects the TME and the latter participates in NED induction in response to castration.

ADT alters the cellular composition of the TME. It activates the PCa immune response and recruits various inflammatory cells for their infiltration (Long et al., 2020). In the early stages of tumor formation, tumor-killing NK and CD8^+^ T cells as well as M1-TAMs are the primary infiltrating inflammatory cells and inhibit tumor cell proliferation (Zhong et al., 2021). In the later stages of tumor formation, however, the infiltrating inflammatory cells are transformed into various inhibitory inflammatory cells that secrete IL-8, IL-6, and TGF-β, which promote NEPC formation (Jia et al., 2014; Long et al., 2020).

ADT directly modulates the non-cellular components of the TME and promotes NEPC formation. ADT may trigger oxidative stress, activate NF-κB signaling, and upregulate stem cell TFs (Yu et al., 2018). The synthesis of certain miRNAs depends on ARE2 in the promoter region. Thus, stable androgen signaling and ADT regulate NEPC formation by modulating miRNA secretion (Zhang et al., 2020; Zhao et al., 2020). ADT also upregulates glucose-regulated protein 78 (GRP78) in tumor cells and triggers miR29-b-mediated downregulation of secreted protein acidic and rich in cysteine (SPARC). This glycoprotein is secreted by adjacent stromal cells in response to ADT, upregulates glucose-regulated protein 78 (GRP78), and triggers miR29-b-mediated SPARC downregulation, which upregulates IL-6 and induces NED (Enriquez et al., 2021).

### Neuroendocrine prostate cancer therapy targeting the tumor microenvironment: Present and future

The TME may play a vital role in NEPC formation. Therefore, targeting various TME components is a potential NEPC therapy.

Targeted TME therapy should reduce immune cell recruitment and infiltration and reactivate the tumor-killing capacity of infiltrating immune cells in the TME. This process may involve targeted HMGB1 inhibition (Wang et al., 2018), COX-2, IL-6 (Heusinkveld et al., 2011), which promotes TAM differentiation into the M1 subtype and reduces M2-TAM and MDSC infiltration. Certain signaling pathways required to activate VEC proliferation and angiogenesis overlap with NEPC formation. Hence, anti-angiogenesis-related therapy may also inhibit NEPC formation. Nevertheless, the inhibition of blood vessel formation may cause localized tumor hypoxia and paradoxically promote NEPC formation.

NED may also be prevented by directly blocking TME-related downstream molecular signaling pathways. IL-6/STAT3 signaling plays an essential role in EMT. The administration of siltuximab to inhibit STAT3 can block the occurrence of EMT (Davies et al., 2018).

The miRNAs secreted by exosomes are signaling molecules with diverse functions. Certain miRNAs may either promote or inhibit NEPC formation. Future research should endeavor to determine how to inhibit the secretion of miRNAs promoting NEPC formation while promoting the secretion of miRNAs inhibiting NEPC formation.

## Conclusion

The TME enhances PCa cell stemness, induces cell lineage transformation, and promotes NEPC formation. The present review summarized the roles of the cellular and non-cellular components of the TME in NEPC evolution, growth, and progression. Clarification of the roles of the TME may help refine the NEPC formation model, aid in the discovery of novel NEPC treatment options targeting the TME, and provide new directions for clinical NEPC treatment.

## Summary

In the present review, we introduce and summarize the roles that the tumor microenvironment (TME) plays in regulating and inducing neuroendocrine prostate cancer (NEPC) formation through various pathways. This synopsis demonstrates the contribution of the TME in refining the model of NEPC formation and lays the foundation for discovering novel NEPC therapies targeting the TME.
